# Interaction between Cannabinoid System and Toll-Like Receptors Controls Inflammation

**DOI:** 10.1155/2016/5831315

**Published:** 2016-08-11

**Authors:** Kathleen L. McCoy

**Affiliations:** Department of Microbiology and Immunology, Virginia Commonwealth University, P.O. Box 980678, Richmond, VA 23298-0678, USA

## Abstract

Since the discovery of the endocannabinoid system consisting of cannabinoid receptors, endogenous ligands, and biosynthetic and metabolizing enzymes, interest has been renewed in investigating the promise of cannabinoids as therapeutic agents. Abundant evidence indicates that cannabinoids modulate immune responses. An inflammatory response is triggered when innate immune cells receive a danger signal provided by pathogen- or damage-associated molecular patterns engaging pattern-recognition receptors. Toll-like receptor family members are prominent pattern-recognition receptors expressed on innate immune cells. Cannabinoids suppress Toll-like receptor-mediated inflammatory responses. However, the relationship between the endocannabinoid system and innate immune system may not be one-sided. Innate immune cells express cannabinoid receptors and produce endogenous cannabinoids. Hence, innate immune cells may play a role in regulating endocannabinoid homeostasis, and, in turn, the endocannabinoid system modulates local inflammatory responses. Studies designed to probe the interaction between the innate immune system and the endocannabinoid system may identify new potential molecular targets in developing therapeutic strategies for chronic inflammatory diseases. This review discusses the endocannabinoid system and Toll-like receptor family and evaluates the interaction between them.

## 1. Introduction

*Cannabis sativa*, better known as marijuana, has been used in traditional medicine for millennia to treat various ailments [1–4]. Development of cannabinoids from the cannabis plant as therapeutic agents has been hindered by their recreational abuse and addictive properties [5, 6]. Legalization of medical marijuana is a growing trend during the past few years. Medical marijuana is primarily used to treat glaucoma and to stimulate appetite and prevent weight loss in AIDS and cancer patients [1–4]. At present, the Food and Drug Administration has approved two cannabinoid medications, Marinol® containing a psychoactive phytocannabinoid and Cesamet® consisting of a synthetic cannabinoid, for the treatment of nausea, emesis, and cachexia [6–8]. In several other countries, Sativex® has been approved to treat spasticity and neuropathic pain in multiple sclerosis patients and pain in patients with advanced cancer [1, 9]. It contains an equimolar combination of psychoactive Δ^9^-tetrahydrocannabinol and nonpsychoactive cannabidiol, which are phytocannabinoids [1, 9]. Nevertheless, the beneficial health effects of cannabinoids, for the most part, remain empirical and anecdotal. However, discovery of two major cannabinoid receptors and endogenous cannabinoids in humans provides the opportunity to understand the mechanisms of action and to develop approaches for manipulating the cannabinoid system as effective treatment for particular human diseases.

Familiar effects of marijuana result from phytocannabinoids acting as neurotransmitters or modulating neurotransmitter release that, in turn, causes euphoria, diminished pain, altered sensory perception, impaired memory, and enhanced appetite [6, 7]. Neurological consequences of cannabinoid exposure can be attributed to cannabinoid receptors within the brain. Moreover, cannabinoids impact other biological systems besides the central nervous system. Numerous studies report that cannabinoids suppress* in vitro* functions of human and animal immune cells, and animals exposed to cannabinoids have decreased host resistance to various pathogens and tumors (reviewed in [10–12]). Chronic cannabis use is associated with increased incidence of rhinitis, pharyngitis, asthma, bronchitis, and sexually transmitted diseases [5, 9, 13, 14]. Besides social behavior contributing to an increased rate of sexually transmitted diseases, depressed immune functions could enhance susceptibility of marijuana users to infections [5].

Because all immune cells examined so far express cannabinoid receptors regardless of their cell lineage, all types of immunity are sensitive to cannabinoid modulation [10–12]. The importance of the cannabinoid system in regulating immune competency is revealed by altered immune status in mice genetically deficient in cannabinoid receptors [15]. In terms of adaptive immunity, cannabinoids usually suppress primary antibody responses to T cell-dependent antigens, induction of cytotoxic CD8^+^ T cells, and cytokine production by helper CD4^+^ T cells, whereas other adaptive immune responses are unaffected or enhanced [10–12]. The current view is that cannabinoid exposure skews T cell responses leading to suppression of cell-mediated immunity and inflammatory reactions [10–12]. Furthermore, cannabinoids impact innate immunity that mediates inflammatory responses and promotes initiation of adaptive immune responses. For example, alveolar macrophages isolated from chronic marijuana users have compromised phagocytosis of microorganisms, ability to kill bacteria, and production of proinflammatory cytokines [16, 17]. These consequences of drug use parallel* in vitro* cannabinoid suppression of immune functions by monocytes, macrophages, and macrophage cell lines of human and rodent origins [10–12]. My laboratory reported that cannabinoids impair the ability of murine macrophages to function as antigen-presenting cells resulting in depressed helper CD4^+^ T cell responses [18–20]. Furthermore, macrophages from mice lacking cannabinoid receptor expression are refractory to cannabinoid suppression of antigen-presenting cell function [21, 22]. Therefore, cannabinoids can exert their influence on an immune response before helper CD4^+^ T cell activation.

The endocannabinoid system consists not only of cannabinoid receptors, but also of endogenous cannabinoids and their biosynthetic and metabolizing enzymes. Macrophages are major producers of endogenous cannabinoids [23], which may not be a coincidence. Both exogenous and endogenous cannabinoids inhibit proinflammatory cytokine production by macrophages stimulated through Toll-like receptors (TLRs). TLRs play a crucial role in macrophages sensing danger to trigger inflammatory responses. Conversely, activation of macrophages via TLRs alters their expression of cannabinoid receptors and levels of endogenous cannabinoids. This review discusses the endocannabinoid system and TLR family and evaluates the interaction between them with emphasis on the innate immune system.

## 2. Endocannabinoid System

### 2.1. Cannabinoid Receptors

Cannabinoid receptors encompass multiple subtypes (reviewed in [24–26]). Central cannabinoid receptor type 1 (CB1) and peripheral cannabinoid receptor type 2 (CB2) are the predominant receptors and share approximately 44% homology [27–30]. Endogenous cannabinoids also bind Transient Receptor Potential Vanilloid 1 receptor, a capsaicin receptor, which is structurally different from CB1 and CB2 receptors [24–26]. The orphan receptor GPR55 may be another receptor subtype, although it has low homology to the other cannabinoid receptors [24–26]. Other candidate receptors have been implicated by pharmacological and functional studies [24–26]. CB1 and CB2 receptors greatly differ in their tissue distribution. CB1 receptor, originally identified in rat cerebral cortex, is primarily expressed in the central nervous system [27, 28]. This receptor subtype is also expressed in various peripheral tissues, such as testis, vascular endothelium, and small intestine [27, 28]. Its expression is heterogeneous within the nervous system and is mainly responsible for cannabinoid psychoactive properties. In contrast, CB2 receptor was originally identified in the promyelocytic leukemic cell line HL60 and is prevalent within the immune system [29, 30]. All lineages of immune cells express the CB2 receptor, although its expression level varies among the cell types. In rank order, B cells express the highest level followed by natural killer cells, macrophages, monocytes, polymorphonuclear cells, and T cells [31]. CB2 receptor expression in healthy brains is limited to a few neurons in the brain stem [32, 33]. However, during neurological diseases, such as multiple sclerosis and Alzheimer's disease, microglial cells, which are brain macrophages, express a high level of CB2 receptor [33, 34]. Some immune cells, including monocytes, also express the CB1 receptor [35–37]. When both receptor subtypes are present in immune cells, the CB2 receptor is usually expressed at a significantly higher level than the CB1 receptor [35–37]. Unlike CB1 receptor-mediated cell activation, signal transduction through the CB2 receptor lacks psychotropic effects [38, 39] making it an attractive target for immunotherapy.

### 2.2. Exogenous and Endogenous Cannabinoids

Cannabis is a complex mixture of over 100 cannabinoids along with other classes of compounds that have pharmacological and biological activities. Numerous synthetic analogues have been produced based on structure-activity relationship studies [24, 25]. Many synthetic cannabinoid analogues have biological effects similar to their natural counterparts [40, 41]. Cannabimimetic compounds are lipophilic molecules and are classified into four main groups based on their chemical structure [24, 25].

The classical group consists of dibenzopyran derivatives, and the phytocannabinoids fall into this group [24, 25]. Δ^9^-Tetrahydrocannabinol is the major psychoactive compound in marijuana and the best-studied cannabinoid. It is a partial agonist binding both CB1 and CB2 receptors and is an ingredient of Marinol and Sativex. Another notable member is cannabidiol that is a nonpsychoactive ingredient of Sativex. Cannabidiol does not activate cannabinoid receptors and yet has biological activity, including immune suppression. Instead, cannabidiol appears to behave as a potent antagonist or inverse agonist [24, 25]. Cannabigerol is the common biosynthetic precursor of Δ^9^-tetrahydrocannabinol and cannabidiol [26]. Major homologues of Δ^9^-tetrahydrocannabinol and cannabidiol are Δ^9^-tetrahydrocannabivarin and cannabidivarin, respectively, with propyl side chains rather than pentyl side chains [26]. Other members are synthetic analogues of phytocannabinoids, and some synthetic compounds are selective receptor agonists with higher affinity for one or other cannabinoid receptor subtype.

Nonclassical cannabinoids are bicyclic and tricyclic analogues of Δ^9^-tetrahydrocannabinol [24, 25]. These synthetic compounds lack a pyran ring. Several are selective cannabinoid receptor agonists. The best-studied member is (−)-cis-3-[2-hydroxy-4-(1,1-dimethylheptyl)phenyl]-trans-4-(3-hydroxypropyl) cyclohexanol referred to as CP55,940, which is a full nonselective agonist with higher potency than Δ^9^-tetrahydrocannabinol.

Cannabinoids belonging to the aminoalkylindole group are very structurally different from classical and nonclassical compounds [24, 25]. They were initially developed as potential analogues of nonsteroidal anti-inflammatory drugs [42]. The most widely studied member is (R)-(+)-[2,3-dihydro-5-methyl-3-(4-morpholinylmethyl)pyrrolo [1,2,3-de]-1,4-benzoxazin-6-yl]-1-napthalenylmethanone called WIN 55,212-2 with slightly higher CB2 than CB1 receptor affinity.

Eicosanoids are the endogenous compounds and are oxidized derivatives of 20-carbon fatty acids [24, 25]. Arachidonoyl-ethanolamide also called anandamide was the first one isolated from porcine brain and was found to have cannabimimetic activity [43]. Anandamide is an arachidonic acid derivative that is highly sensitive to oxidation and hydrolysis. It behaves as a partial agonist and has lower intrinsic activity for CB2 than for CB1 receptor. Another prominent member is 2-arachionoyl glycerol, a monoglyceride, which is more potent than anandamide. Initially, 2-arachionoyl glycerol was isolated from intestine and brain [44, 45], and its concentration in the brain is approximately 170-fold higher than that of anandamide [46]. Because 2-arachionoyl glycerol favors the CB2 receptor, it is viewed as the main endocannabinoid to modulate immune functions. Several synthetic analogues of anandamide have been produced to achieve higher potency and efficacy.

Most cannabinoids exhibit stereoselectivity in pharmacological assays due to chiral centers in the molecules. Frequently, stereoselectivity is also observed in biological assays. Classical and nonclassical cannabinoids and aminoalkylindoles are far more active than their corresponding enantiomer or stereoisomer [24, 25]. Stereoselectivity is one criterion for receptor-mediated actions.

### 2.3. Cannabinoid Signal Transduction Pathways

**Figure 1:**
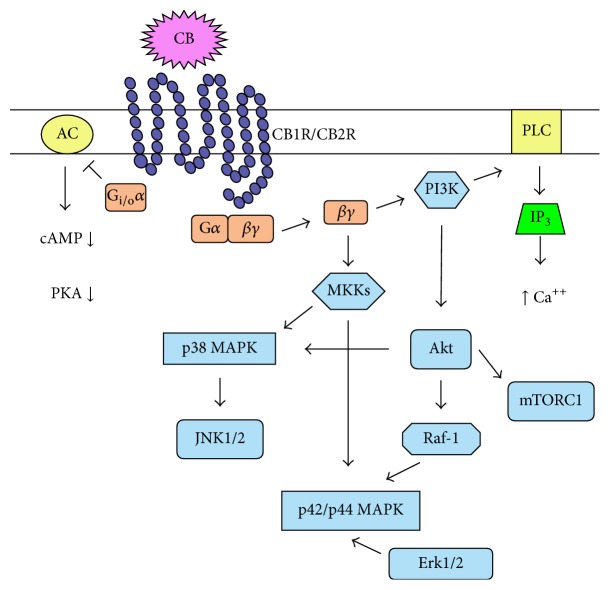
Cannabinoid receptor signal transduction pathway. Upon cannabinoid receptor engagement by a cannabinoid, the activated G_i/o_ *α* subunit inhibits adenylate cyclase activity causing a decrease in cAMP, which, in turn, decreases PKA activity. The *βγ* dimer activates PI3K, which, in turn, activates PLC that ultimately leads to increased intracellular calcium levels. PI3K can active the MAPK pathways. Akt may lead to mTORC1 activation. The *βγ* dimer can, also, activate MKK leading to activation of the MAPK pathways. CB: cannabinoid; CB1R: cannabinoid type 1 receptor; CB2R: cannabinoid type 2 receptor; AC: adenylate cyclase; PKA: protein kinase A; PI3K: phosphatidylinositol-3 kinase; PLC: phospholipase C; IP_3_: inositol trisphosphate; mTORC1: mammalian target of rapamycin complex 1; MKK: mitogen-activated protein kinase kinases; MAPK: mitogen-activated protein kinase; JNK: Jun kinases; Erk: extracellular signal-regulated kinases.

Cannabinoid receptors are seven-transmembrane-spanning G protein-coupled receptors [27–30]. The first evidence that cannabinoid receptors are G_i/o_ protein-coupled receptors was cannabinoid-induced inhibition of adenylate cyclase leading to decreased intracellular cAMP level [47]. Pertussis toxin inhibition of a cannabinoid effect confirms G_i/o_ protein-mediated signal transduction [48]. G proteins are heterotrimers, and upon activation the *α* subunit dissociates from the *βγ* dimer (Figure 1). The G_i/o_ *α* subunit inhibits adenylate cyclase decreasing intracellular cAMP levels [40, 41]. In turn, cAMP-dependent protein kinase A activity diminishes leading to less active transcription factor cAMP response element-binding protein affecting gene expression [40, 41]. The G protein *βγ* dimer eventually leads to activation of the mitogen-activated protein kinase (MAPK) pathways and phosphatidylinositol-3 kinase (PI-3K) [49, 50].

MAPK and Akt regulation by cannabinoid receptor signaling pathways is not well understood (Figure 1). PI-3K inhibitors attenuate cannabinoid-induced activation of MAPK in Chinese hamster ovary cells transfected with human CB1 receptor cDNA [50]. This finding indicates that PI-3K leads to Akt activation eventually causing p42/44 and p38 MAPK activation. Similarly, Δ^9^-tetrahydrocannabinol activates the PI-3K/Akt pathway in epithelial cells leading to Raf-1-mediated activation of p42/p44 MAPK [41]. In contrast, stimulation of rat microglial cells with 2-arachionoyl glycerol causes MAPK activation that is dependent on protein kinase C, not PI-3K [51]. Conversely, WIN 55,212-2 has the opposite effect and inhibits p42/p44 MAPK activation in murine splenic immune cells [52]. Akt may activate mammalian target of rapamycin (mTOR) present in complex 1 (mTORC1), and, in turn, mTORC1 regulates protein synthesis, glucose metabolism, and autophagy. Rapamycin, a mTOR inhibitor, blocks cannabinoid-induced neural progenitor cell proliferation and cannabinoid-enhanced oligodendrocyte differentiation [53, 54]. On the other hand, WIN 55,212-2 decreases mTORC1 activation in prostate cancer cells [55]. Furthermore, stimulation of promyelocytic HL60 cells with 2-arachionoyl glycerol or other cannabinoid agonists does not activate Akt or mTOR [40]. Thus, cannabinoids may or may not activate Akt, MAPKs, and mTOR depending on the cannabinoid group type, cannabinoid concentration, and/or cell type with differential cannabinoid receptor expression.

While CB1 and CB2 receptors share many signaling steps, distinct differences have been identified in the signaling pathways of theses receptors. CB1 receptor signaling can cause increased intracellular Ca^+2^ level, which may result from phospholipase C activation [40]. Stimulation through the CB1 receptor also activates A-type and inwardly rectifying potassium channels and inhibits N- and P/Q-type calcium currents in neural cells [40, 41, 49]. Most notably, CB1 receptor signaling may increase intracellular cAMP levels and cAMP-dependent protein kinase A activity due to the receptor associating with G_s_ proteins [40]. Furthermore, the CB1 receptor may associate with G_q_ proteins leading to phospholipase D activation [40]. While the CB1 receptor may couple to G_s_ or G_q_ proteins, the CB2 receptor does not [41].

### 2.4. Endocannabinoid Biosynthesis and Metabolism

Within the central nervous system, anandamide and 2-arachionoyl glycerol are not preformed molecules stored in vesicles but rather are synthetized when cells are stimulated, such as depolarization of neurons [56, 57]. Their synthesis depends on increased intracellular Ca^+2^ concentration mainly due to Ca^+2^ dependency of the biosynthetic enzymes [58–61]. Anandamide is predominantly produced in a two-step enzymatic process starting with phosphatidylethanolamine that is catalyzed by Ca^+2^-dependent N-acyltransferase followed by N-acylphosphatidylethanolamine-hydrolyzing phospholipase D (reviewed in [62–65]). The critical precursors of 2-arachionoyl glycerol are* sn*-1-acyl-2-arachidonoylgylerols, and multiple enzymatic pathways generate 2-arachionoyl precursors [63]. The main pathway involves phosphoinositide-selective phospholipase C or similar phospholipases followed by two* sn*-1-selective diacylglycerol lipase isoenzymes (reviewed in [62–65]). In contrast to anandamide, various stimuli in addition to Ca^+2^-mobilization trigger 2-arachionoyl glycerol synthesis in neural cells, epithelial cells, and macrophages [56, 64]. Unlike anandamide, 2-arachionoyl glycerol is an important precursor of other molecules [63], which may explain their differential resting levels. In most cases, enhanced anandamide and 2-arachionoyl glycerol biosynthesis is limited in time and location, and, thus, their release affects only cells in the nearby vicinity.

After release, endocannabinoids are rapidly internalized into cells by an undefined mechanism, and a proposed transporter has not been definitely identified. Intracellular anandamide is principally hydrolyzed by fatty acid amide hydrolase, an integral plasma membrane protein [66]. Similarly, internalized 2-arachionoyl glycerol is primarily hydrolyzed by monoacylglycerol lipase associated with the plasma membrane [67]. Reaction products do not activate cannabinoid receptors and may recycle back into their respective biosynthetic pathways [63]. One hydrolysis product, arachidonic acid, may be metabolized by various enzymes to produce prostaglandins, thromboxanes, leukotrienes, and other biologically active compounds, which are potent inflammatory mediators or immune suppressors. Anandamide and 2-arachionoyl glycerol themselves may be oxidized by cytochrome P450, cyclooxygenase-2, and 5- and 12-lipoxygenases [63, 65]. Some lipoxygenase products bind both CB1 and CB2 receptors [25, 56, 57]. Hence, results from metabolic enzyme inhibitors or enzyme deficient mice should not presume to be caused by only increased endocannabinoid levels.

## 3. Toll-Like Receptor Family

A fundamental characteristic of the immune system is the ability to distinguish between self and non-self-molecules or antigens. Immune cells are particularly adept at detecting microbial antigens, and the subsequent immune response can clear an infection and provide protection against a future infection. Innate immunity represents the first line of defense against infectious diseases, and cells, such as neutrophils and macrophages, are the first responders. Inflammation is the initial immune response against infectious agents, and the inflammatory response promotes initiation of an adaptive immune response by antigen-specific T and B cells. However, cells of innate immunity do not express antigen-specific receptors, unlike T and B cells of adaptive immunity. Decades ago, Janeway proposed a hypothesis that innate immune cells utilize germline-encoded receptors that are antigen-selective [68], and such receptors were eventually identified many years later. Innate immune cells express pattern-recognition receptors that bind conserved molecular patterns, and engagement of these receptors transduces a signal allowing cells to sense danger in the form of a pathogen or host cellular damage [69, 70]. These receptors are present at the cell surface, in endocytic organelles, or in the cytoplasm permitting perception of both extracellular and intracellular dangers. The major receptor gene families based on protein domain homology include TLRs, retinoid acid-inducible gene-1-like receptors, absent in melanoma-2 receptors, C-type lectin receptors, intracellular DNA sensors, and nucleotide-binding domain, leucine-rich repeat-containing receptors (or nucleotide-binding, oligomerization domain-like receptors) that are discussed in several comprehensive reviews [70–76]. This review focuses on the TLR family, which has been extensively investigated and was the first one discovered.

### 3.1. Toll-Like Receptor Family Members

Toll gene was first identified in* Drosophila* as essential in regulating embryonic development of dorsal-ventral polarity [77, 78]. In addition, Toll has a critical role in resistance to fungal infections in adult flies [79]. This latter discovery was key to understanding innate inflammatory triggers and led to finding the mammalian homologues [80, 81]. Although the number of family members varies among mammalian species, TLRs are evolutionarily conserved type I transmembrane proteins [70–76]. TLRs are predominantly expressed by innate immune cells, especially dendritic cells and macrophages, while adaptive immune B and T cells, and nonimmune cells, including fibroblasts and epithelial cells, have limited TLR expression [70–76]. Family members have a highly conserved cytoplasmic Toll/Interleukin-1 receptor (TIR) domain that initiates signal transduction via recruitment of adaptor proteins [70–76]. At present, 10 TLR chains have been identified in humans, while TLR10 is a pseudogene in mice, but three additional chains (TLR11 to TLR13) are expressed in mice [70–76]. Each TLR recognizes a distinct set of molecular patterns [70–76]. Promiscuous ligand recognition, which is determined by the leucine-rich repeat extracellular domain, is indispensable for the handful of TLRs to mediate effective immune defenses against an array of diverse pathogens.

### 3.2. Exogenous and Endogenous Ligands

Regarding mammalian host resistance, TLRs bind pathogen-associated molecular patterns (PAMPs) present within microbial molecules, but absent from mammalian molecules [70–75]. The broad gamut of ligand specificity ranges from hydrophobic lipids to hydrophilic nucleic acids. TLRs are divided into two subfamilies based on their cellular location, and the following discussion will focus on the TLR chains expressed in humans. TLR1 and TLR2, TLR4 through TLR6, and TLR10 are plasma membrane proteins, whereas TLR3 and TLR7 through TLR9 reside in membranes of endocytic organelles. Interestingly, ligand recognition correlates with the cellular location of TLRs [70–75]. Cell surface TLRs sense fungal cell wall components (TLR2), bacterial lipopolysaccharides (TLR4), lipoproteins (TLR1, TLR2, and TLR6), flagella (TLR5), or peptidoglycans (TLR2). On the other hand, intracellular TLRs are specific for viral double-stranded RNA (TLR3), single-stranded RNA (TLR7 and TLR8), DNA (TLR9), or unmethylated CpG DNA (TLR9). In contrast, human TLR10 is an orphan receptor without a known ligand. Receptor compartmentalization influences the types of ligands accessible for cell activation. For example, endosomal TLRs engage when virulent intracellular pathogens infect cells, but do not signal when the pathogen remains outside the cell. Although all TLRs possess a similar TIR domain, cell surface and intracellular receptors, in general, utilize different adaptor molecules to transduce a signal [70–75]. Cell surface TLR signaling mainly relies on TIR-containing adaptor protein (TIRAP) and protein myeloid differentiation primary response 88 (MyD88). Furthermore, TIR domain-containing adaptor-inducing interferon-*β* (TRIF) and TRIF-related adaptor molecule (TRAM), also, transmit TLR signals. The different pairs of adaptor molecules activate distinct transcription factors, and, hence, influence the nature and outcome of the inflammatory response.

For many years, one puzzling aspect of innate immunity has been inflammatory responses in the absence of an infection that contribute to tissue damage during autoimmune diseases and chronic inflammatory diseases. Matzinger proposed that the immune system does not distinguish between self and non-self-antigens per se but rather is designed to detect danger [82]. The apparent paradox of sterile inflammation is resolved by realization that pattern-recognition receptors, including TLRs, recognize endogenous ligands. The endogenous molecules called damage-associated molecular patterns (DAMPs) are created or released upon tissue injury or cell death [69, 76]. Heat shock protein 60 was the first candidate DAMP reported to induce TLR4 activation [83]. Within months, necrotic cells were shown to induce proinflammatory gene expression and dendritic cell maturation via TLR2 [84, 85]. Since then, several DAMPs have been identified, and some are intracellular molecules to which innate immune cells are not normally exposed. Analogous to PAMPs, cell surface and intracellular TLRs recognize different types of DAMPs, although endogenous ligands have not been identified for all TLRs [69, 76]. For example, TLR2 and TLR4 ligands include heat shock proteins, serum amyloid A, and oxidized low-density lipoprotein. Intracellular TLR3 recognizes mRNA, and TLR9 binds antibody-chromatin complexes. Recent evidence indicates that danger signals provided by PAMPs and DAMPs synergistically activate inflammatory responses.

### 3.3. MyD88-Dependent Signal Transduction Pathway

Functional TLRs form dimers and particular receptors associate with a coreceptor and/or an accessory molecule [69–76, 86]. Most TLRs form homodimers with TLR2 as the notable exception pairing with TLR1 or TLR6. These TLR2 heterodimers have different ligand specificities. When a ligand binds, the TIR domains oligomerize recruiting adaptor proteins that initiate downstream signaling events.

**Figure 2:**
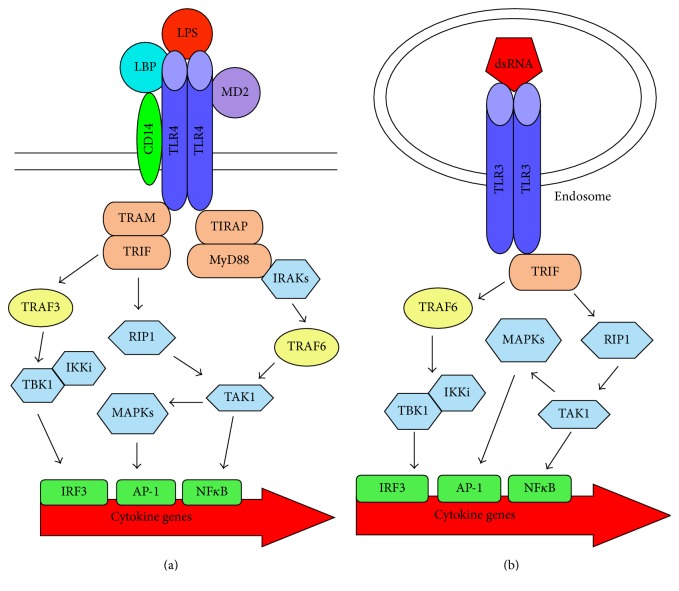
TLR signal transduction pathways. (a) Signaling through TLR4 by bacterial LPS. TLR4 forms a complex with LBP, MD2, and CD14. When LPS binds TLR4, two adaptor complexes are recruited. The MyD88-dependent pathway activates IRAKs eventually leading to activation of TAK1 that leads to activation of transcription factor NF-*κ*B and stimulates the MAPK pathways for transcription factor AP-1 activity. The TRIF-dependent pathway occurs within endosomes and activates RIP1, also resulting in TAK1 activation. Through TRAF3, IKKi and TBK1 are activated leading to the activation of transfer transcription factor IRF-3. (b) Signal through TLR3 by nucleic acids occurs within endosomes. When the receptor is occupied, TLR3 alone binds TRIF without TRAM. RIP1 is activated leading to the same subsequent events as those during TLR4 signaling. TRIF also activates IKKi and TBK1 leading to transcription factor IRF-3 activation, but involves TRAF6, not TRAF3. TLR: Toll-like receptor; LPS: lipopolysaccharide; LBP: LPS-binding protein; MD2: myeloid differentiation-2 protein; MyD88: myeloid differentiation primary response 88; TIR: Toll/interleukin-1 receptor; TIRAP: TIR-containing adaptor protein; TRIF: TIR domain-containing adaptor-inducing interferon-*β*; TRAM: TRIF-related adaptor molecule; IL: interleukin; IRAK: IL-1R-associated kinases; TNF: tumor necrosis factor; TRAF: TNF receptor-associated factors; TBK1: TANK-binding kinase-1; MAPK: mitogen-activated protein kinase; RIP1: receptor-interacting serine/threonine-protein kinase 1; TAK1: tumor growth factor-*β*-activated kinase 1; IKKi: inducible I*κ*B kinase-I; IRF-3: interferon regulatory factor-3; AP-1: activator protein-1; NF-*κ*B: nuclear factor-*κ*B.

TLR4, the prototype and best-studied receptor, is unique among TLRs using four adaptor molecules. The classic TLR4 ligand is lipopolysaccharide (LPS) from Gram-negative bacteria, which causes septic shock. The LPS response requires a molecular complex (Figure 2(a)) consisting of TLR4 homodimer, LPS-binding protein, CD14, and myeloid differentiation-2 protein (MD-2) [69–76]. MD-2 is required for TLR4 dimerization. Upon LPS exposure, the TLR4:MD-2 complex concentrates within cholesterol-rich lipid rafts containing CD14, which is anchored to the plasma membrane by glycosylphosphatidylinositol. The LPS-binding protein, a soluble serum protein that binds lipid A moiety, initiates sequential transfer of LPS monomers to coreceptor CD14 and then to MD-2. Gioannini and Weiss estimate that LPS monomers extracted from one bacterium are sufficient to activate 1,000 macrophages [87]. Hence, sequential LPS transfer is thought to heighten sensitivity of innate immune cells, in particular macrophages.

Adaptor TIRAP binds the TIR domain of TLR4 but lacks a signaling domain [70–75, 86]. TIRAP itself contains a TIR domain that subsequently recruits adaptor MyD88 to the TLR4 complex (Figure 2(a)). The MyD88-dependent signaling pathway triggers IL-1R-associated kinases and MAPKs culminating in activation of transcription factors, nuclear factor- (NF-) *κ*B, and activator protein-1. These transcription factors induce nitric oxide and proinflammatory cytokine production.

All TLRs except TLR3 utilize MyD88, and the other cell surface receptors, also, interact with TIRAP similar to TLR4 and lead to NF-*κ*B activation [69–76, 86]. Similar to TLR4, TLR2 heterodimers localize to lipid rafts and require MyD88 via TIRAP for signal transduction. However, TLR2/TLR1 activation involves coreceptor CD14, whereas CD36 serves as the coreceptor for TLR2/6 [70, 88]. CD36 functions as a scavenger receptor in monocytes and macrophages and participates in phagocytosis and endocytosis [89]. CD36 might mediate TLR2/6 internalization to downregulate cell activation.

### 3.4. TRIF-Dependent Signal Transduction Pathway

Unlike other TLRs, TLR4 signals involve a second pair of adaptor proteins resulting in type I interferon production (Figure 2(a)) and a second wave of NF-*κ*B activation [69–76, 86]. In this pathway, adaptor TRAM and TRIF molecules drive cell activation. Analogous to TIRAP, TRAM lacks a signaling domain and recruits a second adaptor molecule, in this case TRIF, to the plasma membrane. TRIF recruits signaling molecules that, in turn, recruit inducible I*κ*B kinase-i and TANK-binding kinase-1 to the complex. Ultimately, transcription factor interferon regulatory factor- (IRF-) 3 becomes activated leading to type I interferon synthesis, especially interferon-*β*. Receptor-interacting protein kinase-1 activity eventually leads to TRIF-dependent MAPKs and NF-*κ*B activation.

Both TIRAP and TRAM localize to the plasma membrane; however their binding to TLR4 appears to be mutually exclusive [90]. Colocalization, time kinetics, and endocytosis inhibition studies indicate that TRAM engages after TLR4 internalization [70, 72–75, 86]. A novel model of TLR4 signaling proposes that CD14 mediates trafficking of TLR4 to the endosomes, whereupon the TRIF-dependent pathway is induced [70, 72–75, 86]. Hence, the MyD88-dependent and TRIF-dependent signaling pathways are sequestered from each other, and commence at distinct cellular locations.

### 3.5. Intracellular TLR Signal Transduction Pathways

Intracellular TLRs stimulate cells by divergent pathways compared with TLR4 [70, 72–75, 86]. Intracellular TLR7 to TLR9 do not utilize the TRAM/TRIF pathway. Instead, engagement of these receptors induces type I interferon production via IRF7 and NF-*κ*B activation in a MyD88-dependent manner. The alternative MyD88 pathway triggers IL-1R-associated kinases to activate transcription factors IRF7 and NF-*κ*B. On the other hand, TLR3 employs the adaptor TRIF protein (Figure 2(b)) but does not need either TRAM or MyD88. The high affinity TIR domain of TLR3 directly binds TRIF in the absence of TRAM. Notably, a point mutation within the TIR domain of TLR3 switches its specificity from TRIF to MyD88 [91]. Similar to TLR4, this TRIF-dependent pathway activates transcription factor IRF3 via TANK-binding kinase-1 inducing robust interferon-*β* secretion.

Cell activation by TLR signals is tightly regulated on multiple levels. Critical regulatory mechanisms range from TLR trafficking and cleavage to protein modification of signaling molecules. Furthermore, negative regulators are important in preventing autoimmune and inflammatory diseases. TLR recognition of PAMPs is crucial for host resistance to infectious diseases, and individuals with defective TLR responses are immune compromised. On the flip side, abnormal TLR activation by PAMPs, mutations in TLR signaling molecules, and TLR activation by DAMPs are associated with the development and pathogenesis of numerous diseases, including autoimmune diseases, hypersensitivities, chronic inflammatory diseases, cancer, and cardiovascular diseases. Hence, cannabinoid modulation of TLR signal transduction pathways may have beneficial or detrimental consequences on human health.

## 4. Interaction between Cannabinoid and Toll-Like Receptor Activation

Important innate immune cells expressing TLRs are monocytes, macrophages, microglial cells, and dendritic cells. These myeloid cells are closely related. Monocytes circulate in the blood and mature into macrophages or dendritic cells depending on the stimulus. Microglial cells are resident macrophages in the brain. All these cells express cannabinoid receptors, and cannabinoids influence their immune functions. Of note, alveolar macrophages isolated from chronic marijuana users are compromised in secreting proinflammatory cytokines, such as tumor necrosis factor-*α* and interleukin-6, in response to LPS [16, 17]. This review discusses cannabinoid modulation of TLR ligand responses by innate immune cells, and vice versa.

### 4.1. Impact of Cannabinoids on Toll-Like Receptor Responses

Cannabinoid studies regarding TLRs have concentrated on bacterial LPS responses via TLR4 as a classic model for inflammation (Table 1). For the most part, exogenous and endogenous cannabinoids interfere with proinflammatory cytokine and nitric oxide production by LPS- or LPS/interferon-*γ*-stimulated monocytes, macrophages, microglia, and macrophage cell lines in culture [10–12, 92, 93]. However, one study reported increased interleukin-1*β* secretion by LPS-activated resident peritoneal cells caused by Δ^9^-tetrahydrocannabinol [94] in opposition to other investigations [95, 96]. Cannabinoids display biphasic dose-response curves for cytokine secretion in some culture systems [96, 97], which may account for this apparent discrepancy. Interestingly, Δ^9^-tetrahydrocannabinol decreases LPS-induced cyclooxygenase-2 expression in mouse macrophage J774 cell line [93], which would diminish endocannabinoid 2-arachidonoyl-glycerol metabolism, thereby augmenting immune suppression. In addition, chronic marijuana use increases CB1 and CB2 receptor expression on peripheral blood monocytes [98], which may enhance cannabinoid sensitivity. Thus, exogenous cannabinoids may alter the endocannabinoid system leading to greater suppression of the LPS response.

Cannabinoids directly impair TLR-induced cell activation in culture (Table 1). Δ^9^-Tetrahydrocannabinol perturbs posttranslational processing of tumor necrosis factor-*α* protein in LPS-activated mouse macrophages [99, 100]. Furthermore, cannabinoids diminish proinflammatory cytokine production in LPS-stimulated mouse microglial BV-2 cell line that is accompanied by decreased transcription factor NF-*κ*B activity and corresponding cytokine mRNA levels [101]. LPS stimulates nitric oxide production and release through induction of nitric oxide synthase-2 expression under NF-*κ*B regulation. Similar to cytokine suppression, cannabinoids attenuate nitric oxide release, nitric oxide synthase-2 gene expression and enzymatic activity, and transcription factor NF-*κ*B activation in LPS- or LPS/interferon-*γ*-activated myeloid cells [92, 102–104]. Analogously, cannabinoids suppress peptidoglycan-stimulated cell growth of a glioma cell line via TLR2 with concomitant decreased NF-*κ*B activation [105]. These results indicate that cannabinoids directly interfere with TLR signal transduction.

On the other hand, cannabinoids may also indirectly suppress* in vitro* cytokine production (Table 1). For example, a CB2 receptor-selective agonist prevents LPS-upregulated TLR4 expression on mouse bone marrow dendritic cells rendering the cells less LPS responsive [106], although a similar cannabinoid effect upon TLR4 expression has not been reported for other innate immune cells. Moreover, cannabinoids induce apoptosis that has been proposed as an immunosuppressive mechanism [107, 108]. However, the majority of findings regarding cannabinoid inhibition of TLR-mediated responses cannot be attributed to apoptosis or cell toxicity. Lastly, cannabinoids may induce other immune suppressive processes. Anandamide and a CB2 receptor-selective agonist induce production of interleukin-10, an inhibitory cytokine, in LPS/interferon-*γ*-activated myeloid cells [109, 110]. CP55,940 induces interleukin-1 receptor antagonist expression in LPS-stimulated microglial cells [111], which interferes with interleukin-1 signals. Thus, multiple modes of action may mediate cannabinoid suppression of* in vitro* LPS responses.

LPS administration in animals is frequently used as* in vivo* models of inflammation and bacterial sepsis (Table 2). A synthetic cannabinoid diminishes LPS-stimulated proinflammatory cytokine levels in the brain and blood of rats [112]. In the LPS-induced pulmonary inflammation model, exogenous and endogenous cannabinoids dose-dependently decrease tumor necrosis factor-*α* level in bronchoalveolar fluid and reduce neutrophil infiltration into the lungs in mice [113]. Synthetic cannabinoids rescue* C. parvum*-primed mice from LPS lethality and diminish serum proinflammatory cytokine levels [114]. Likewise, a nonpsychoactive synthetic cannabinoid abolishes LPS-induced hypotensive response in rats and rescues mice from the lethal effects of LPS and D-galactosamine coadministration [115]. A CB2 receptor-selective agonist also protects against mortality and acute liver failure, decreases proinflammatory cytokines levels, and increases inhibitory interleukin-10 level in mice given LPS and D-galactosamine  [116]. Upon* in vitro* LPS stimulation, cytokine and nitric oxide production by macrophages from mice previously given Δ^9^-tetrahydrocannabinol remain suppressed without additional drug in the cultures [102]. Perhaps, cannabinoid immune suppression induced* in vivo* may persist after drug removal. Therefore, the effects of cannabinoids on LPS activation in several animal models, in general, parallel the* in vitro* findings.

Involvement of cannabinoid receptors in suppressing TLR responses has been established by multiple approaches. One set of criteria is cannabinoid receptor-selective agonists causing inhibition and cannabinoid receptor-selective antagonists reversing inhibition. Cannabinoid suppression of proinflammatory cytokine and nitric oxide production is CB2 receptor-mediated in LPS-stimulated cultured cells, LPS-induced pulmonary inflammation, and LPS/galactosamine-induced acute liver failure [113, 116] (Table 2). On the other hand, protection of* C. parvum*-primed mice from LPS lethality and diminished NF-kB activity in TLR2 ligand-stimulated glioma cells are mediated through the CB1 receptor [105, 114], whereas both cannabinoid receptors participate in decreasing cytokine levels after* in vivo* LPS administration [112]. Mice genetically deficient in cannabinoid receptor expression are the best evidence for receptor participation. CB2 receptor-deficient mice are highly susceptible to induced inflammatory diseases, including contact dermatitis, experimental autoimmune encephalomyelitis, atherosclerosis, and carbon tetrachloride-induced liver damage [15, 117]. Incidence and severity of the diseases are exacerbated in the CB2 receptor-deficient mice compared to wild-type mice [15, 117]. Importantly, the CB2 receptor-deficient mice have lower survival, more pronounced tissue damage, and increased serum interleukin-6 levels in a sepsis model [118]. Mice lacking both cannabinoid receptors have markedly heightened allergic inflammation leading to exacerbated contact dermatitis, delayed-type hypersensitivity, and inflammatory responses to* Influenza* virus [119–121]. Alveolar macrophages and bone marrow dendritic cells from mice deficient in both cannabinoid receptors have a more mature phenotype, have increased expression of major histocompatibility complex class I and class II molecules, and are more efficient in activating T cells [120, 121], suggesting that the absence of endocannabinoid signals may alter differentiation and maturation of innate immune cells towards hyperresponsiveness. Perhaps, the role of the CB1 receptor is more readily detected in* in vivo* models due to interactions among various cell types. The absence of both cannabinoid receptors has a more dramatic impact on the immune system, indicating the possible interplay of cannabinoid receptors during disease processes, which has important implications in developing cannabinoids as therapeutic agents for inflammatory diseases.

Microglial cells play an important role in neuroinflammation and appear to be a special case in terms of cannabinoid receptor-mediated immune suppression. General consensus is that resting microglial cells express a low level of the CB1 receptor and lack CB2 receptor expression [12, 56, 57]. However, microglial cells from diseased tissues or microglial cells activated in culture gain CB2 receptor expression [12, 56, 57]. Criteria used for cannabinoid receptor involvement in diminished proinflammatory cytokine secretion and nitric oxide release from LPS-activated microglial cells in culture reveal all possible outcomes encompassing both cannabinoid receptors, or the CB1, CB2, or no cannabinoid receptor [103, 109, 111, 123–125]. A receptor-independent mechanism despite cannabinoid receptor expression on the cells implies that cannabinoid receptor expression is too low to exert a biological effect, or the receptors are inactive. A receptor-independent mechanism may involve disruption of lipid rafts due to the hydrophobicity of cannabinoids [141]. As discussed above, lipid rafts are critical for proper assembly of the TLR4 and TLR2 complexes, and their disruption would contribute to a decreased TLR response. Differences in agonist or antagonist concentrations, and cell activation state among the studies, may contribute to disparate findings concerning cannabinoid receptor participation. Additional investigation is needed to resolve this issue.

Cannabinoids exert immune suppression when innate immune cells are activated, but not when the cells are resting or quiescent. When direct suppression is cannabinoid receptor-mediated, the two signal transduction pathways (Figures 1 and 2(a)) would cross-talk. Transcription factor NF-*κ*B is activated through the MyD88-dependent signal transduction pathway via both TLR4 and TLR2. Hence, cannabinoids must interfere with the MyD88-dependent signal transduction pathway to decrease NF-*κ*B activity along with diminishing cytokine production and cell growth through TLR4 and TLR2. In some cases, cell permeable cAMP to counteract the active G_i/o_ subunit reverses cannabinoid inhibition of cytokine secretion and nitric oxide release [10–12, 102, 124] suggesting the involvement of decreased protein kinase A activity in mediating suppression. However, protein kinase A activity is not necessary for cytokine gene expression in TLR-activated cells (Figure 2). Other studies suggest cannabinoid regulation of p42/p44 MAPK activation participates in immune suppression [41, 52]. Activation of the MAPK pathways is required for cytokine gene transcription. In this scenario, too much of a positive signal becomes negative. Excessive MAPK activation may generate a negative feedback loop. Although a remaining question is what is the link between the cannabinoid and TLRs signaling pathways, MAPKs are attractive candidates.

Cannabinoids augment interferon-*β* production during the TLR3 ligand response (Table 1). Very few studies have examined the impact of cannabinoids on TLR3 signal transduction and interferon-*β* production (Figure 2(b)), and, thus, studies with nonimmune cells are discussed below. Interferon-*β* is a type I interferon with antiviral and anti-inflammatory activities and is a treatment for multiple sclerosis patients [142]. WIN 55,212-2 does not suppress but rather enhances interferon-*β* mRNA expression in polyinosinic:polycytidylic acid-activated HEK293 cell line transfected with TLR3 cDNA [127]. Interferon-*β* transcript upregulation is accompanied by increased MAPK and transcription factor IRF3 activities; however tumor necrosis factor-*α* secretion and transcription factor NF-*κ*B activity decrease in the TLR3-transfected cells [127]. Analogously, WIN 55,212-2 augments interferon-*β* mRNA expression but decreases tumor necrosis factor-*α* secretion in polyinosinic:polycytidylic acid-activated primary astrocytes and peripheral blood mononuclear cells from multiple sclerosis patients [126, 127]. Perhaps, excessive MAPK activation leads to decreased NF-*κ*B activity as discussed for the LPS response.The opposing effects on IRF3 and NF-*κ*B activities indicate that the cannabinoid alters a signaling step downstream of TRIF binding TLR3.

In striking contrast, WIN 55,212-2 has the opposite effect on interferon-*β* mRNA and transcription factor IRF3 in TLR4-transfected HEK293 cells stimulated with LPS [126] (Table 1). Likewise, Δ^9^-tetrahydrocannabinol and nonpsychoactive cannabidiol inhibits interferon-*β* mRNA expression and protein secretion in LPS-activated mouse microglial BV-2 cell line [125]. An antagonist of peroxisome proliferator-activated receptor-*α* blocks the enhanced interferon-*β* level without affecting tumor necrosis factor-*α* level in TLR3-transfected HEK293 cells [127], indicating sensitivity of transcription factor IRF3, but not NF-*κ*B, activity to peroxisome proliferator-activated receptor-*α*. However, sensitivity of interferon-*β* inhibition to the peroxisome proliferator-activated receptor-*α* antagonist was not examined in TLR4-transfected HEK293 cells. The opposing cannabinoid effects on interferon-*β* induced via TLR3 versus TLR4 signaling pathways (Figure 2) raise the question of why peroxisome proliferator-activated receptor-*α* does not enhance interferon-*β* production in LPS-stimulated cells exposed to cannabinoids, unless the receptor is not activated during TLR4 signaling. The disparate cannabinoid effect on interferon-*β* production implies two different molecular targets in the TLR3 and TLR4 pathways. Perhaps, the TRAM:TRIF and TRIF adaptors, or TRAF3 and TRAF6 used by TLR4 and TLR3, respectively, have differential sensitivity to cannabinoid immune modulation.

### 4.2. Impact of Toll-Like Receptor Activation on Endocannabinoid System

Cannabinoid receptor expression by immune cells varies depending on the cell type, maturational stage, and activation state. Innate immune cells have a high degree of plasticity, and their cannabinoid receptor expression can be manipulated intentionally (Table 3). Human peripheral blood monocytes, human dendritic cells, and some monocyte/macrophage cell lines constitutively express both CB1 and CB2 receptors [129, 138, 143]. Maturation of human peripheral blood monocytes and monocytic THP-1 cells and differentiation of human promyelocytic HL60 cell line into macrophages by phorbol esters upregulate cannabinoid receptor expression [143–145]. Notably, Δ^9^-tetrahydrocannabinol impairs LPS-mediated differentiation of human monocytes into dendritic cells [122]. On the other hand, resident mouse macrophages lack detectable CB1 and CB2 receptor mRNA, whereas inflammatory thioglycollate-elicited macrophages express a high level of the CB2 receptor, and interferon-*γ* stimulation may further increase CB2 receptor expression [128]. Microglial cells express no/low level of CB1 receptor mRNA, and CB2 receptor mRNA is undetectable [128, 129, 146]. Stimulation of rodent microglial cells with interferon-*γ* induces CB2 receptor mRNA expression [129, 130]. The level of cannabinoid receptor expression on immune cells affects cannabinoid immune modulation. For example, a nonpsychoactive cannabinoid inhibits LPS-induced interleukin-6 expression only after human monocytes mature into macrophages [147]. Hence, particular stimuli induce or upregulate cannabinoid receptor expression during myeloid cell differentiation, maturation, and activation affecting their sensitivity to cannabinoids.

**Figure 3:**
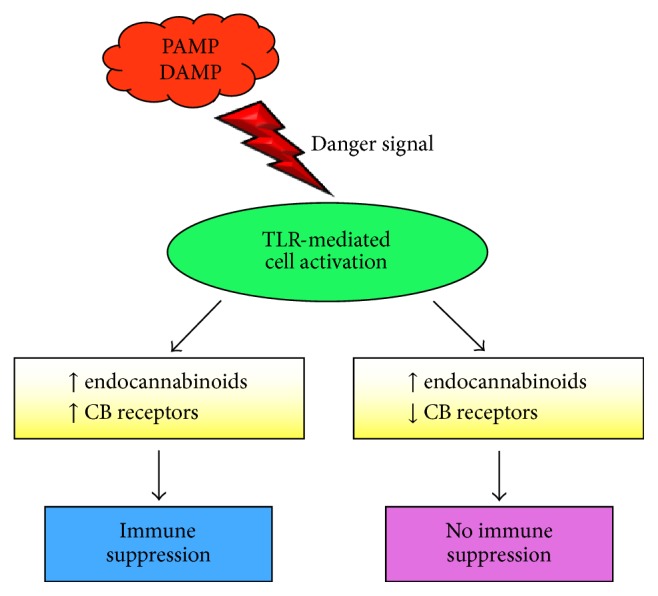
Interaction between TLR-mediated activation and endocannabinoid system. PAMPs and DAMPs provide dangers signals that activate innate immune cells via TLRs. Activated cells increase endocannabinoid levels that may suppress the inflammatory response. Cell activation may increase or decrease cannabinoid receptor expression. Increased receptor expression in the presence of endocannabinoids promotes immune suppression. Decreased receptor expression may render cells resistant to cannabinoid-mediated immune suppression, and the inflammatory response continues. PAMPs: pathogen-associated molecular patterns; DAMPs: damage-associated molecular patterns; TLR: Toll-like receptors; CB: cannabinoid.

The influence of only TLR4 signal transduction has been investigated on the endocannabinoid system in myeloid cells (Table 3). LPS modulation of cannabinoid receptor expression in myeloid cells depends on the experimental system (Figure 3). For example, CB2 receptor mRNA level drops in thioglycollate-elicited macrophages in response to LPS [128, 131]. In contrast, LPS activation of mouse macrophage RAW 264.7 cell line induces CB1 receptor mRNA and upregulates CB2 receptor mRNA expression [129, 132, 139]. Protein kinases A and C inhibitors partially block the LPS effect on CB1 and CB2 receptor expression [132], although involvement of the cAMP-protein kinase A pathway in LPS signaling is controversial [148]. Likewise,* in vivo* LPS administration to rodents upregulates CB2 receptor expression in microglial cells [132, 133]. Importantly, various pathological conditions are associated with altered cannabinoid receptor expression, usually increased CB2 receptor expression [10, 57]. Hence, the differential LPS impact on cannabinoid receptor expression may reflect plasticity of innate immune cells to cues from their microenvironment.

Evidence is growing that endocannabinoid levels change during various disease processes due to altered catalytic activities of the biosynthetic or metabolizing enzymes. In several animal disease models, modified endocannabinoid levels exert pro- or anti-inflammatory effects on innate immune cells based on enzyme inhibitors, transporter inhibitors, and mice genetically deficient in the enzymes. This research area is discussed in detail elsewhere [56, 57, 92, 133, 149].

Danger signals provided to innate immune cells increase endocannabinoid production (Figure 3). LPS administration* in vivo* decreases metabolizing fatty acid amide hydrolase activity in mouse peripheral blood mononuclear cells [134], which would increase anandamide levels (Table 3). Similarly, LPS administration also diminishes 2-arachidonyl glycerol hydrolytic activity within the spleen and liver [135]. Anandamide is barely detected in rat monocytes and macrophage J774 cell line, whereas the cells contain substantial anandamide levels upon LPS stimulation [136, 140]. In addition, LPS activation of rat macrophages and J774 cells also increases 2-arachidonyl glycerol levels due to decreased 2-arachidonyl glycerol hydrolytic activity in the cells [137]. Human immature dendritic cells contain enhanced 2-arachidonyl glycerol, but not anandamide, levels upon LPS activation [138]. Conversely, low dose-LPS activation of macrophage RAW 264.7 cell line increases anandamide, but not 2-arachidonyl glycerol, levels along with enhanced biosynthetic N-acyltransferase and phospholipase D activities [23, 137]. The rapid time kinetics of augmented anandamide level [23] indicates a direct effect of the TLR4 signal transduction pathway as opposed to autocrine stimulation by secreted cytokines. In support of this possibility, increased anandamide level in RAW 264.7 cells is prevented by MAPK and NF-*κ*B inhibitors [23], indicating involvement of the MyD88-dependent signal transduction pathway (Figure 2(a)). Although what endocannabinoids increase during LPS stimulation appears to depend on the cell type and experimental conditions, higher endocannabinoid levels are accompanied by increased biosynthetic enzyme activity and/or decreased metabolizing enzyme activity.

## 5. Conclusions

When the immune system encounters a pathogen, innate immune cells recognize the pathogen via TLRs and other pattern-recognition receptors to trigger an inflammatory response. Innate immune cells are an important source of endocannabinoids, and these cells synthesize and metabolize endocannabinoids. TLR-mediated activation of the innate immune cells enhances their endocannabinoid levels (Figure 3). In the absence of an infection, tissue damage produces DAMPs perceived as danger signals via TLRs to activate innate immune cells. Indeed, local endocannabinoid production increases in response to tissue damage during disease progression and infections [150]. Abundant evidence demonstrates that cannabinoids have anti-inflammatory activity, which is the desired consequence during sterile inflammation. Considering that 2-arachidonyl glycerol behaves as a chemoattractant [129, 145], locally enhanced endocannabinoid levels may recruit immune cells to the site and mitigate further tissue damage. Thus, innate immune cells may play a role in regulating endocannabinoid homeostasis, and, in turn, the endocannabinoid system modulates local inflammation. TLR signals also alter cannabinoid receptor expression by innate immune cells, which affects their sensitivity to cannabinoids. During progression of an inflammatory disease, cells may become refractory to cannabinoid immune suppression despite elevated endocannabinoid levels, and the inflammatory response continues and may intensify. Therefore, the final outcome may be enhanced clearance of an infection, facilitation of tissue healing, or exacerbation of tissue damage. Although definition of the link between cannabinoid and TLR signaling pathways awaits further studies, identification of promising molecular targets may provide insights into therapeutic modalities to control injurious inflammation.

## Figures and Tables

**Table 1:** Cannabinoid effects on *in vitro* TLR responses.

Cells	Stimulus	Effect	Reference
Alveolar macrophages	LPS	↓ inflammatory cytokines	Baldwin et al. [16]
			Pacifici et al. [17]

Monocytes	LPS	↓ inflammatory cytokines	Zurier et al. [95]
		Impairs differentiation to dendritic cells	Roth et al. [122]

Thioglycollate-elicited macrophages	LPS	↓ tumor necrosis factor-*α* protein processing	Zheng and Specter [99]
		↑ interleukin-1*β*	Zhu et al. [94]
	LPS/interferon-*γ*	↓ nitric oxide synthase-2	Coffey et al. [102]
		↓ nitric oxide	Mestre et al. [92]
		↑ interleukin-10	Correa et al. [109]

Blood mononuclear cells	LPS	↓ interleukin-1*α*	Watzl et al. [96]
		Biphasic dose-response	Berdyshev et al. [97]

Microglial cells	LPS	↓ cytokine mRNA	Puffenbarger et al. [103]
		↑ IL-1 receptor antagonist	Molina-Holgado et al. [111]
		↓ nitric oxide	Merighi et al. [123]
	LPS/interferon-*γ*	↑ interleukin-10	Correa et al. [110]
		↓ nitric oxide	Waksman et al. [124]

Dendritic cells	LPS	↓ TLR4 expression	Xu et al. [106]

BV-2 cells	LPS	↓ NF-*κ*B activity	More et al. [101]
		↓ cytokine mRNA	
		↓ interferon-*β* mRNA	Kozela et al. [125]

J774 cells	LPS	↓ nitric oxide	Chang et al. [93]
		↓ interleukin-6	
		↓ cyclooxygenase-2	

RAW 264.7 cells	LPS	↓ tumor necrosis factor-*α* protein processing	Fischer-Stenger et al. [100]
		↓ nitric oxide synthase-2	Jeon et al. [104]
		↓ nitric oxide	
		↓ NF-*κ*B activity	

TLR4-transfected HEK293 cells	LPS	↓ interferon-*β* mRNA	Downer et al. [126]
		↓ IRF3 activity	

Glioma U87MG cells	Peptidoglycan	↓ NF-*κ*B activity	Echigo et al. [105]

TLR3-transfected HEK293 cells	Polyinosinic:polycytidylic acid	↑ interferon-*β* mRNA	Downer et al. [127]
		↑ IRF3 activity	Downer et al. [126]
		↓ tumor necrosis factor-*α*	
		↓ NF-*κ*B activity	

Blood mononuclear cells	Polyinosinic:polycytidylic acid	↑ interferon-*β* mRNA	Downer et al. [126]
		↓ tumor necrosis factor-*α*	

**Table 2:** Cannabinoid effects on *in vivo* TLR responses.

Animal model	Cell or tissue	Effect	Reference
LPS	Blood & brain	↓ inflammatory cytokines	Roche et al. [112]
		CB1R & CB2R-mediated	
	Cardiovascular system	↓ hypotensive response	Gallily et al. [115]
	Blood	↓ tumor necrosis factor-*α*	
		↑ survival	

LPS-induced pulmonary inflammation	Bronchoalveolar fluid	↓ tumor necrosis factor-*α*	Berdyshev et al. [113]
	Lungs	↓ neutrophil infiltration	
		CB2R-mediated	

*C. parvum*/LPS	Blood	↓ inflammatory cytokines	Smith et al. [114]
		↑ survival	
		CB1R-mediated	

D-Galactosamine/LPS	Liver	↓ inflammatory cytokine	Tomar et al. [116]
		↑ interleukin-10	
		↓ acute liver failure	
		↓ cell infiltration	
		CB2R-mediated	
		↑ survival	Gallily et al. [115]

Sepsis in CB2R^−/−^ mice	Blood	↑ interleukin-6	Tschöp et al. [118]
	Lungs	↑ tissue damage	
		↓ survival	

CB2R^−/−^ mice		↑ incidence & severity of induced inflammatory various diseases	Reviewed in Buckley [15] & Malfitano et al. [117]

CB1R/CB2R^−/−^ mice		↑ contact dermatitis	Karsak et al. [119]
		↑ delayed-type hypersensitivity	
		↑ *Influenza*-induced inflammation	Buchweitz et al. [120]
	Alveolar macrophages	More mature phenotype	Karmaus et al. [121]
	Dendritic cells	More mature phenotype	

**Table 3:** Inflammatory effects on endocannabinoid system.

Cell or tissue	Stimulus *in vitro* or animal model	Effect	Reference
Macrophages	Thioglycollate *in vivo*	↑ CB2 receptor	Carlisle et al. [128]

Microglial cells	Interferon-*γ*	Induces CB2 receptor	Walter et al. [129]
			Maresz et al. [130]

Thioglycollate-elicited macrophages	LPS	↓ CB2 receptor	Carlisle et al. [128]
			Cabral et al. [131]

Microglial cells	LPS *in vivo*	↑ CB2 receptor	Mukhopadhyay et al. [132]
			Concannon et al. [133]

Blood mononuclear cells	LPS *in vivo*	↓ fatty acid amide hydrolase activity	Wolfson et al. [134]

Spleen & liver	LPS *in vivo*	↓ 2-arachidonyl glycerol hydrolytic activity	Szafran et al. [135]

Monocytes	LPS	↑ anandamide	Varga et al. [136]

Macrophages	LPS	↑ 2-arachidonyl glycerol	Pestonjamasp and Burstein [137]
		↓ 2-arachidonyl glycerol hydrolytic activity	

Dendritic cells	LPS	↑ 2-arachidonyl glycerol	Matias et al. [138]

RAW 264.7 cells	LPS	Induces CB1 receptor	Walter et al. [129]
		↑ CB2 receptor	Mukhopadhyay et al. [132]
			Friedman et al. [139]
		↑ anandamide	Liu et al. [23]
		↑ N-acyltransferase	Pestonjamasp and Burstein [137]
		↑ phospholipase D	

J774 cells	LPS	↑ anandamide	Di Marzo et al. [140]
		↑ 2-arachidonyl glycerol	Pestonjamasp and Burstein [137]
		↓ 2-arachidonyl glycerol hydrolytic activity	
